# The Preoperative Fasting Conundrum: An Audit of Practice in a Tertiary Care Children’s Hospital

**DOI:** 10.5152/TJAR.2022.21132

**Published:** 2022-06-01

**Authors:** Sujata Shivlal Rawlani, Nandini Malay Dave, Priyanka Pradeep Karnik

**Affiliations:** Department of Paediatric Anaesthesia, NH-SRCC Children’s Hospital, Mumbai, India

**Keywords:** Audit, children, fasting guidelines, non-adherence, preoperative fasting

## Abstract

**Objective:**

This article aimed to study preoperative fasting times in children undergoing elective surgery and to analyze the effect of active interventions conducted to promote compliance with current fasting guidelines.

**Methods:**

An initial audit was performed in which 85 children up to 15 years of age posted for elective surgeries were surveyed. A questionnaire was circulated among nurses, resident medical officers, and surgeons to assess their knowledge regarding recent fasting guidelines and its importance. The mean preoperative fasting times were found to be much longer than the recommended guidelines. Interventions were carried out to spread awareness about recent preoperative fasting guidelines. A re-audit was done 4 months after the initial audit.

**Results:**

The initial audit revealed a mean preoperative fasting time for solids and water to be 9.43 hours and 6.64 hours, respectively. About 43.6% of hospital staff believed “fasting from midnight” regimen is the best method to prevent pulmonary aspiration. Incorrect orders by doctors (47%) and ward nurses (38%) were found to be important causes of non-adherence. After the intervention, mean preoperative fasting times for solids and water decreased to 7.7 hours and 2.6 hours, respectively.

**Conclusion:**

Adopting simple measures such as education and multidisciplinary teamwork can optimize fasting duration and children’s experiences preoperatively.

**Main Points**

- The “6-4-2” regimen for preoperative fasting is a widely accepted regimen for children undergoing elective surgeries. However, many children have fasted excessively in spite of the guidelines.
- An initial audit was performed in which 85 children up to 15 years of age posted for elective surgeries were surveyed. It revealed that about 13% of children have fasted for more than 12 hours for solids and 27% of children have fasted for more than 8 hours for clear liquids.
- The analysis of the questionnaire (Appendix 1) revealed that 50.8% reported that conflicting instructions given by one or more source was the major factor influencing deviation from standard fasting guidelines in our hospital.
- Awareness programs in the form of lectures were conducted. Information leaflets were put up on bulletins across the hospital to ensure compliance. A well-designed copy of recent fasting guidelines was circulated among all surgeons.
- A re-audit was conducted 4 months later, and it was found that children without food for more than 12 hours decreased from 13% to 6%, and children without clear fluids for more than 8 hours decreased from 27% to 2%.

## Introduction

Pulmonary aspiration after anaesthesia is a rare event. The reported incidence is 0.07%-0.1%.^1^ However, traditional practices and the fear of the theoretical risk of aspiration often lead to very long preoperative fasting times. There is ample evidence now that gastric emptying times are different for solids and liquids and that clear liquids pass through the gastric antrum very quickly. This led to the guidelines being reviewed by various anaesthesia societies and revised into 6 hours for solids, nonhuman, and formula milk, 4 hours for breast milk, and 2 hours for clear fluids (6-4-2 regimen)^2^ which is widely accepted.

Preoperative fasting is intended to allow sufficient time for gastric emptying. It is important to realize that although adequate preoperative fasting is mandatory before subjecting a patient to general anaesthesia, equal importance and attention should be paid to prevent excessive fasting, especially in the paediatric population. Children when subjected to prolonged fasting become irritable or fatigued and may also suffer physiological derangements like dehydration, hypoglycemia, and metabolic imbalances. Reduction of fasting period within acceptable safety limits not only improves patient compliance and co-operation but also enhances the quality of care.^3^ This audit was undertaken to identify current practices pertaining to the duration of preoperative fasting in our hospital and to analyze the effect of active interventions conducted to promote compliance with current fasting guidelines.

## Methods

An approval from the hospital ethics committee was obtained (ERR No: R-202016; dated October 2020), and written informed consent for participation in the study was taken from the parents. Fasting times in children up to 15 years of age scheduled for elective surgeries were surveyed for a period of 1 month. A detailed preoperative history was obtained regarding the time of the last intake of solids, breast milk, formula milk, juices, and clear liquids by the child. In addition, the source from whom the preoperative fasting orders were received and details of exact instructions were noted. Children with risk of aspiration, vomiting, gastroesophageal reflux, enteral tube feeding, and gastrointestinal obstruction were excluded.

Simultaneously during the same study period, a survey was conducted among the ward nurses, resident medical officers (RMOs), and heads of every surgical department to assess their knowledge regarding recent fasting guidelines and its importance. A questionnaire was prepared by doing a thorough literature search of web-based electronic databases such as PubMed, Google Scholar, and Embase. This survey questionnaire comprising 8 questions was mailed to 5 expert paediatric anaesthesiologists (Appendix 1). They were asked to rate the relevance of every question on a 4-point scale (1 = not relevant, 2 = somewhat relevant, 3 = relevant, and 4 = highly relevant). Item content validity index (ICVI) for each question was calculated (ICVI = number of experts rating questions as 3-4 divided by a total number of experts). The questions with ICVI >0.78 were retained. Scale content validity index (SCVI) of the questionnaire was 0.96 (SCVI = total of ICVI scores divided by the total number of included questions), which was within acceptable limits.^4^ After the validation, the questionnaire was circulated in the form of Google forms among ward nurses, RMOs, and surgeons.

Subsequent to the audit and survey, the following interventions were carried out. Awareness programs in the form of didactic lectures using PowerPoint presentations were conducted for ward nursing staff, doctors, and RMOs regarding recent fasting guidelines and its importance. The nursing staff was promoted to encourage children to consume clear liquids 2 hours prior to surgery. Written instructions regarding the same were inserted in preanaesthetic charts. Information leaflets were put up on bulletins across the hospital to ensure compliance (Appendix 2). A well-designed copy of recent fasting guidelines was circulated through social media (WhatsApp) among all surgeons. The operation theatre nursing staff was instructed to communicate with the ward nurses in case of any rescheduling of surgery. We conducted a re-audit after 4 months of the initial audit to assess if there were any changes with respect to the duration of preoperative fasting times.

## Statistical Analysis

Data entry was done in MS Excel (version 10.0). Data were summarized and presented systematically in tabular form. The children were grouped into 4 groups: less than 1 year, 1-5 years, 5-10 years, and 10-15 years. Quantitative data (fasting times) are presented as mean and standard deviation. Qualitative data (causes of nonadherence to fasting guidelines) are presented with the help of percentage table. The software used in the analysis was Statistical Package for the Social Sciences 17.0 version (SPSS Inc.; Chicago, IL, USA). GraphPad Prism 6.0 version.

## Results

Eighty-five children met the inclusion criteria and were included in the initial audit. Thirty-three (39%) were males and 52 (61%) were females. The mean age of the patients was 5.04 ± 4.4 years, and the percentage-wise distribution of age is shown in Table 1. The preoperative fasting time for food (solids and milk) ranged between 2 hours and 15 hours (mean ± SD 9.43 ± 2.89 hours). The preoperative fasting time for clear liquids ranged between 2 hours and 10.5 hours (mean ± SD 6.64 ± 2.12 hours).

The survey questionnaire was distributed to a total of 64 hospital workers. This included 38 nurses, 10 RMOs, and 16 surgeons. The analysis of the questionnaire (Appendix 1) distributed to evaluate the knowledge regarding recent fasting guidelines revealed that 36.5% of hospital staff believed preoperative fasting is important to prevent pulmonary aspiration. When asked about the duration of preoperative fasting for solid and breast milk, 87.3% and 91.9%, respectively, responded correctly, 98.4% could correctly differentiate what constitutes category of clear liquids, 31.7% believed that 4 hours of fasting to clear liquids is mandatory before surgery, 85.7% could correctly identify the side effects of prolonged fasting, 43.6% of hospital staff believed that “fasting from midnight” regimen is the best method to prevent pulmonary aspiration, 50.8% reported that conflicting instructions given by one or more source was the major factor influencing deviation from standard fasting guidelines in our hospital.

Various causes of nonadherence to fasting guidelines in the initial audit are shown in Table 2. Incorrect orders by doctors and ward nurses were found to be an important cause of noncompliance. Many of the children were kept fasting from midnight irrespective of the time of surgery. There was no rescheduling of surgery due to inadequate fasting during the audit period.

**Figure 1.**
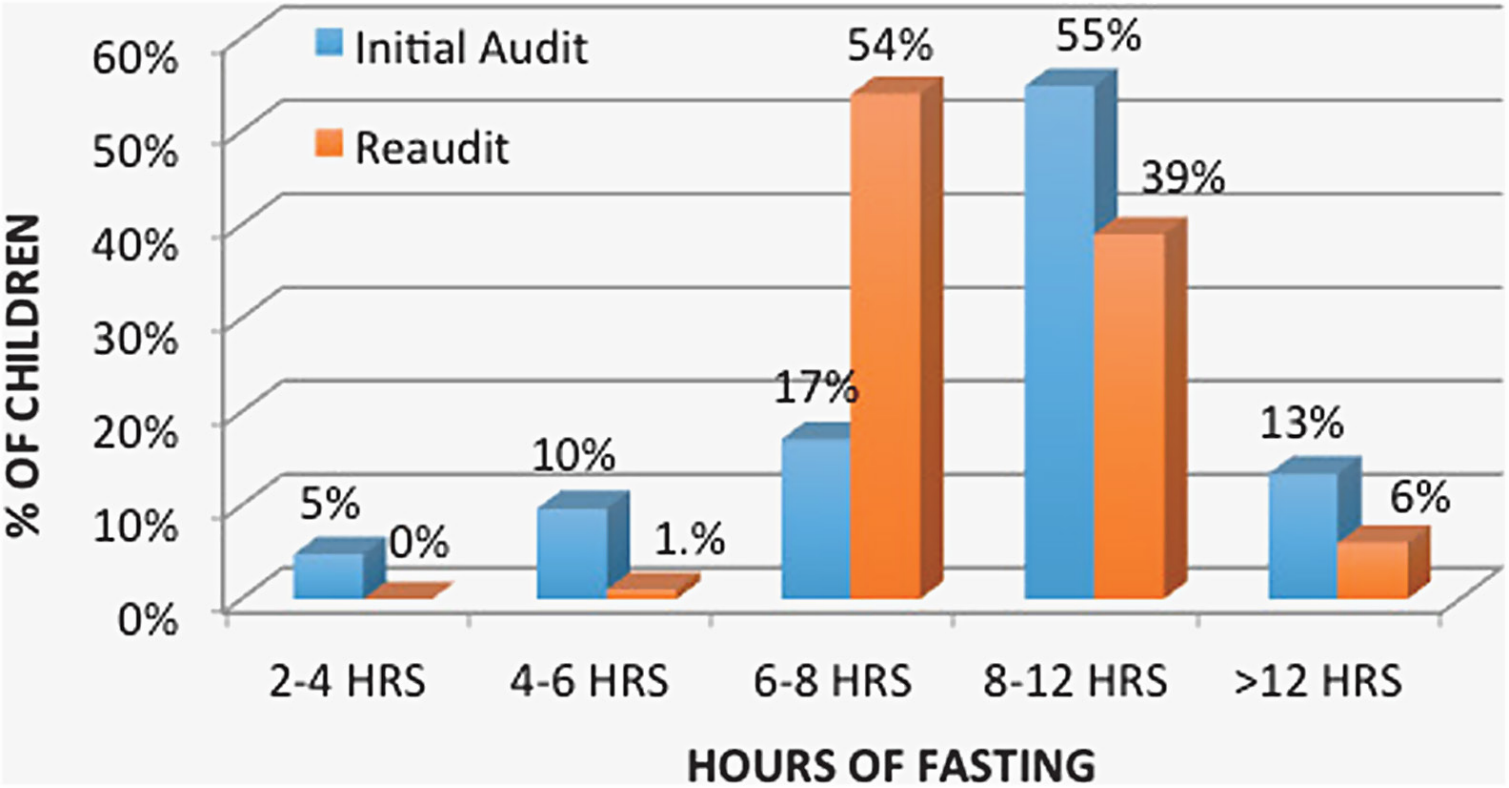
Preoperative fasting times for solids and milk.

Re-audit was done 4 months after the initial audit. A total of 135 children met the inclusion criteria in the re-audit among which 60% were males and 40% were females. The mean age of the patients was 5.5 ± 4.5 years, and the percentage distribution is shown in Table 1. The preoperative fasting time for foods (solids and milk) in the re-audit ranged between 5 hours and 12 hours (mean ± SD 7.77 ± 1.80 hours). Mean preoperative fasting time for food decreased from 9.43 hours to 7.77 hours. Children without food for more than 12 hours decreased from 13% to 6% (Figure 1).

**Figure 2.**
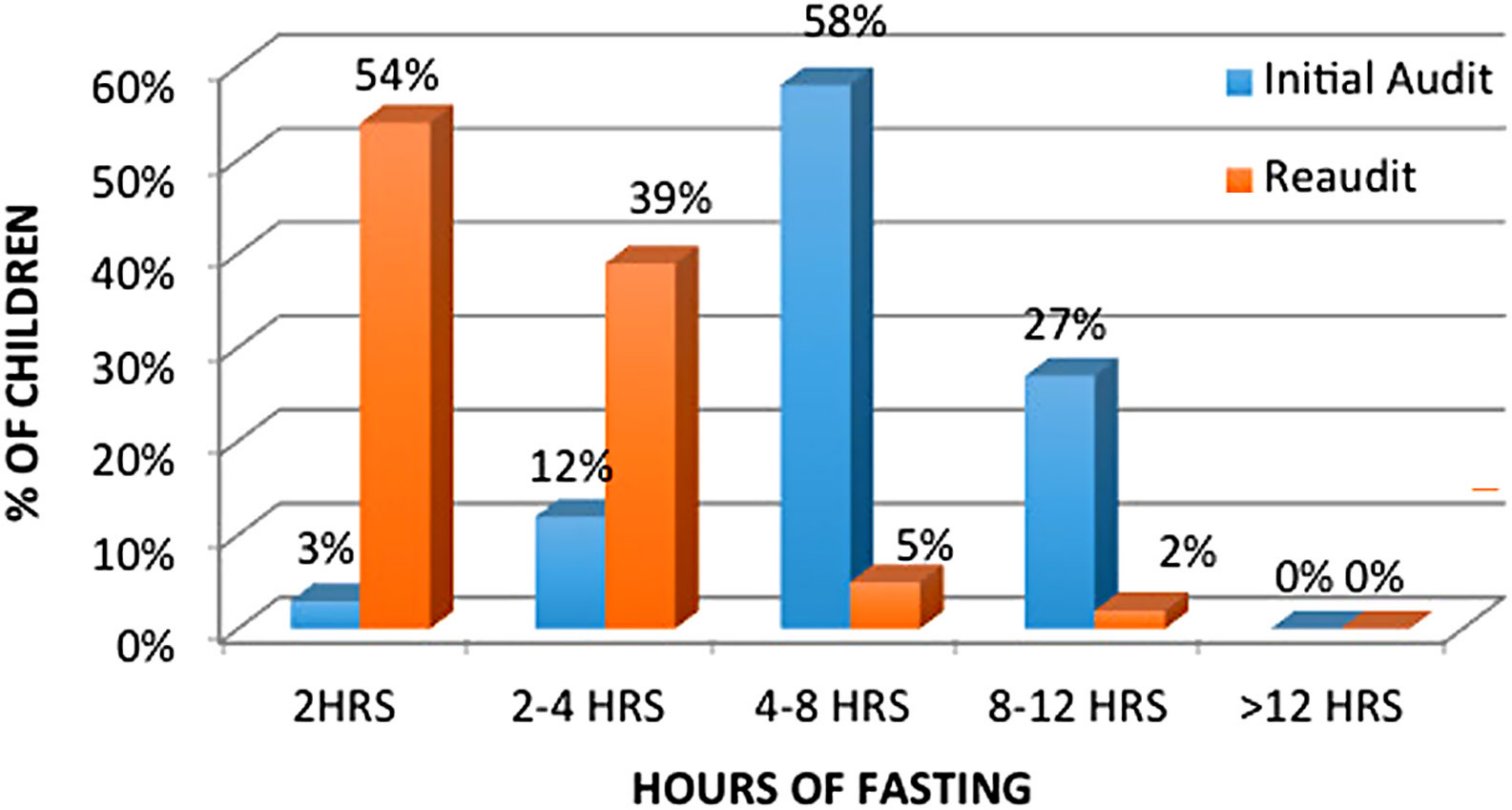
Preoperative fasting times for clear liquids.

The preoperative fasting time for clear liquids in the re-audit ranged between 1 hour and 5 hours (mean ± SD was 2.65 ± 1.00 hours). Mean preoperative fasting time for clear liquids decreased from 6.6 hours to 2.6 hours. Children without clear fluids for more than 8h decreased from 27% to 2% (Figure 2).

## Discussion

Excessive preoperative fasting has been a problem despite established guidelines. We aimed to audit the practice in our hospital and subsequently take corrective measures to promote compliance with standard fasting guidelines.

The results of our initial audit revealed that the mean preoperative fasting time for food (solids and milk) was 9.43 hours and that for clear liquids was 6.64 hours. About 13% of children in our study were found to fast for more than 12 hours for solids and 27% for more than 8 hours for clear fluids. Engelhardt et al^5^ in their study of 1350 children found that children presenting for elective outpatient surgery were fasted excessively resulting in a considerable amount of preoperative discomfort. Their study demonstrated that the median (range) fasting times were 12:05 hours (00:45 hours-21:50 hours) and 07:57 hours (00:05 hours-20:50 hours) for solids and fluids, respectively.

In our initial audit, we tried to find out the causes of nonadherence to fasting guidelines and we found that incorrect orders by doctors followed by incorrect orders by nurses remain the most common cause of prolonged fasting at our hospital. Singla et al^6^ in their study of factors affecting preoperative fasting instructions in children undergoing daycare surgery found that 71.6% of the parents received instructions from the surgeon who wanted to keep scope for alteration in the operative schedule on the day of surgery and therefore gave longer nil per oral instructions, and many of the surgeons were not aware of the recent fasting guidelines which resulted in incorrect preoperative orders.

Adverse outcomes of prolonged fasting include physiological derangements such as hypoglycemia, dehydration, and electrolyte imbalance. In addition, there is a negative impact on parenteral satisfaction and the quality of care.^7^ The results of our initial audit prompted us to take measures in order to bring changes in our practice. Educational programs were conducted for doctors and nurses to increase the awareness regarding fasting guidelines and its importance. Arun and Korula^8^ reported incorrect fasting instructions by the nursing staff as the major reason for prolonged preoperative fasting in children. We focused on educating ward nurses as they remain the main caregivers in the preoperative area. Clear separate instructions for solids and liquids and instructions in written form on the information leaflets were circulated for better recall.

A re-audit was conducted 4 months after the implementation of the corrective measures. The mean preoperative fasting times for solids and liquids reduced significantly as compared to the initial audit. The results of interventions and how long the effects of these programs last can be quite variable.

In a study at a U.S. medical center, preoperative fasting time was 14 hours for solids and 12 hours for liquids.^9^ A follow-up study conducted at the same institution 4 years later still showed a prolonged preoperative fasting time of 14 hours for solids and 11 hours for clear liquids.^10^ Dennhardt et al^11^ implemented a multi-professional program to reduce real fasting times to intervals close to the 6-4-2 guidelines, and they reported a decrease in mean fasting time from 8.5 hours to 6 hours, and a reduction of “deviation from the guideline >2 hours” from 70% to 8%.

Excessive preoperative fasting has remained a common problem. Inadequate understanding of fasting instructions and its necessity by the parents, concerns about complications like vomiting and aspiration, exaggerated pressure to feed a hungry and crying child, and conflicting instructions given by more than 1 person or source may explain the inconsistency in following the recommended guidelines. Sometimes it is a lack of awareness among doctors/nurses about recent fasting guidelines or the tendency to follow age-old practices, which adds to the problem.

Thomas et al^12^ demonstrated that with a 1-hour clear fluid policy, there is no increased risk of pulmonary aspiration and there was a consensus to revise the “6-4-2” regimen to the “6-4-1” regimen to reduce fluid fasting times. Andersson et al^13^ demonstrated that children could be allowed to consume fluids until taken to the operating area, and this practice did not increase the risk of pulmonary aspiration. The solution to the problem is not quite simple. There are multiple factors responsible for noncompliance. Many a time despite correct orders, it becomes difficult to wake up children at midnight and feed them to adhere to the guidelines. Often, children who are scheduled later on the list will have longer fasting times. Encouraging children to have a drink (of clear liquid) as close to surgery as possible may be the answer to minimizing preoperative fasting. It is essential to maintain good coordination between the anaesthesiologist, surgeon, and the ward nurses to get effective results.

Our study was conducted during COVID times and hence our patient numbers are small. We believe, however, that the sample is reflective of the practice in our hospital. We also think we could implement and bring about change because ours is a relatively small 200-bedded dedicated children’s facility. The greater challenge will be to sustain change. Continuing education for all the stakeholders will go a long way to ensure that good practices are maintained.

## Conclusion

An audit of practice showed that children have fasted for much longer times before surgery than recommended. Active interventions resulted in reducing fasting times for both solids and liquids. Educating the hospital staff and improving communication among nurses, surgeons, and anaesthesiologists can overcome challenges in the implementation of current fasting guidelines.

## Figures and Tables

**Table 1.** Age Distribution of Children

**Age Group (Years)**	**Initial Audit (%)**	**Re-audit (%)**
<1	20	16
1-5	36	38
5-10	22	25
10-15	22	21

**Table 2.** Causes of Nonadherence to Fasting Guidelines

Incorrect orders by doctors	47%
Incorrect ward nurses’ orders	38%
Parents not following nurses’ orders	10%
Rescheduling of surgery	5%

## Appendix 1: Fasting Guidelines for Children Questionnaire

1. Why is pre operative fasting important?
  - To improve wound healing.
  - To decrease the risk of aspiration.
  - To prevent perioperative vomiting.
  - Both b & c
2. How many hours of fasting to solids is required before surgery?
  - 2 hours.
  - 4 hours.
  - 6 hours.
  - From Midnight.
3. How many hours of fasting to clear liquids is required before surgery?
  - 2 hours.
  - 4 hours.
  - 6 hours.
  - From Midnight.
4. What among these is considered as clear liquid?
  - Fruit juice with pulp.
  - Milk.
  - Water.
  - Soup.
5. How many hours of fasting to breast milk is required before surgery?
  - 2 hours.
  - 4 hours.
  - 8 hours.
  - From Midnight.
6. What are the side effects of prolonged fasting in children?
  - Hypoglycemia.
  - Dehydration and electrolyte imbalance.
  - Irritability.
  - All of the above.
7. What are the factors influencing deviation from standard fasting guidelines?
  - Conflicting instructions given by one or more source.
  - Fear of postponement of surgery.
  - Pressure to feed a hungry and crying child.
  - All of the above.
8. Which among these following statements is NOT TRUE?
9. Pulmonary aspiration is a rare event.
10. Solids and liquids have different gastric emptying time.
11. “Fasting from midnight” regimen is the best method to prevent pulmonary aspiration.
12. Prolong fasting increases the need for analgesia and also increases the chances of postoperative nausea vomiting.

## References

[1] Kelly CJ Walker RW . Perioperative pulmonary aspiration is infrequent and low risk in pediatric anesthetic practice. Paediatr Anaesth. 2015;25(1):36 43. 10.1111/pan.12549) 25280003

[2] Smith I Kranke P Murat I et al. Perioperative fasting in adults and children: guidelines from European society of anaesthesiology. Eur J Anaesthesiol. 2011;28(8):556 569. 10.1097/EJA.0b013e3283495ba1) 21712716

[3] Hamid S Pre-operative fasting- a patient centered approach. BMJ Qual Improv Rep. 2014;2(2). 10.1136/bmjquality.u605.w1252) PMC4663816 26734235

[4] Polit DF Beck CT . The content validity index: are you sure you know what’s being reported? Critique and recommendations. Res Nurs Health. 2006;29(5):489 497. 10.1002/nur.20147) 16977646

[5] Engelhardt T Wilson G Horne L Weiss M Schmitz A . Are you hungry? Are you thirsty? - fasting times in elective outpatient pediatric patients. Paediatr Anaesth. 2011;21(9):964 968. 10.1111/j.1460-9592.2011.03573.x) 21489044

[6] Singla K Bala I Jain D Bharti N Samujh R . Parents’ perception and factors affecting compliance with preoperative fasting instructions in children undergoing day care surgery: a prospective observational study. Indian J Anaesth. 2020;64(3):210-215. 10.4103/ija.IJA_794_19) PMC7179783 32346168

[7] Brady M Kinn S Ness V O’Rourke K Randhawa N Stuart P . Preoperative fasting for preventing perioperative complications in children. Cochrane Database Syst Rev. 2009;4(4):CD005285. 10.1002/14651858.CD005285.pub2) 19821343

[8] Arun BG Korula G . Preoperative fasting in children: an audit and its implications in a tertiary care hospital. J Anaesthesiol Clin Pharmacol. 2013;29(1):88 91. 10.4103/0970-9185.105810) 23493776 PMC3590550

[9] Crenshaw JT Winslow EH . Preoperative fasting: old habits die hard. Am J Nurs. 2002;102(5):36 44; quiz 45. 10.1097/00000446-200205000-00033) 12006853

[10] Crenshaw JT Winslow EH . Preoperative fasting duration and medication instruction: are we improving? AORN J. 2008;88(6):963 976. 10.1016/j.aorn.2008.07.017) 19054485

[11] Dennhardt N Beck C Huber D et al. Optimized preoperative fasting times decrease ketone body concentration and stabilize mean arterial blood pressure during induction of anesthesia in children younger than 36 months: a prospective observational cohort study. Paediatr Anaesth. 2016;26(8):838 843. 10.1111/pan.12943) 27291355

[12] Thomas M Morrison C Newton R Schindler E . Consensus statement on clear fluids fasting for elective pediatric general anaesthesia. Paediatr Anaesth. 2018;28(5):411 414. 10.1111/pan.13370) 29700894

[13] Andersson H Zarén B Frykholm P . Low incidence of pulmonary aspiration in children allowed intake of clear fluids until called to the operating suite. Paediatr Anaesth. 2015;25(8):770 777. 10.1111/pan.12667) 25940831

